# Secure Multiparty Quantum Computation for Summation and Multiplication

**DOI:** 10.1038/srep19655

**Published:** 2016-01-21

**Authors:** Run-hua Shi, Yi Mu, Hong Zhong, Jie Cui, Shun Zhang

**Affiliations:** 1 School of Computer Science and Technology, Anhui University, Hefei City, 230601, China; 2 Centre for Computer and Information Security Research, School of Computing and Information Technology, University of Wollongong, Wollongong NSW 2522, Australia

## Abstract

As a fundamental primitive, Secure Multiparty Summation and Multiplication can be used to build complex secure protocols for other multiparty computations, specially, numerical computations. However, there is still lack of systematical and efficient quantum methods to compute Secure Multiparty Summation and Multiplication. In this paper, we present a novel and efficient quantum approach to securely compute the summation and multiplication of multiparty private inputs, respectively. Compared to classical solutions, our proposed approach can ensure the unconditional security and the perfect privacy protection based on the physical principle of quantum mechanics.

Secure Multiparty Computation (SMC)^1^ is an important branch in modern cryptography. Secure Multiparty Summation or Multiplication is a fundamental primitive of SMC that enables multiple parties to jointly compute the summation or multiplication of their respective private inputs without revealing any private input. As we know, Secure Multiparty Summation and Multiplication can be used to build complex secure protocols for other multiparty computations, specially, numerical computations. In addition, there are also lots of other important applications of Secure Multiparty Summation and Multiplication in distributed networks, such as secret sharing, electronic voting, secure sorting, data mining and so on.

On the one hand, there existed some classical protocols for Secure Multiparty Summation^2^,^3^,^4^ and Multiplication^5^,^6^,^7^, which were based on classical cryptography. However, classical cryptography cannot provide the unconditionally secure communications and cannot resist the attack of the quantum computer especially.

On the other hand, quantum cryptography can provide the unconditional security, which is guaranteed by physical principles of quantum mechanics. Since Bennett and Brassard^8^ presented the first quantum key distribution protocol (BB84 protocol), quantum cryptography has been widely studied and rapidly developed. Compared to classical cryptography, the most important advantage is that an eavesdropper can easily be detected by using the characteristics of quantum mechanics. Therefore, a lot of results have been gained, such as quantum key distribution, quantum teleportation, quantum secret sharing, quantum secure direct communication, quantum key agreement, quantum signature and so on. Furthermore, SMC is also studied extensively in quantum cryptography^9^,^10^,^11^,^12^,^13^,^14^.

However, there are only a few quantum protocols for Secure Multiparty Summation. In 2007, Du *et al.*^15^ presented a secure quantum addition module *n* + 1 based on non-orthogonal states, where *n* denoted the number of all parties. In 2010, Chen *et al.*^16^ proposed another secure quantum addition module 2 based on multi-particle entangled states with the trusted third party. However, the module of the two protocols is too small, so that it limits their more extensive applications. Furthermore, the two protocols lack high communication efficiencies due to their bit-by-bit computation and communication. In addition, to the best of our knowledge, there is no any quantum protocol for Secure Multiparty Multiplication.

In this paper, we present a novel quantum approach to systematically and efficiently compute Secure Multiparty Summation and Multiplication, in which the computations of Secure Multiparty Summation and Multiplication are securely translated into the computations of the corresponding phase information by the quantum Fourier transform, and later the phase information is extracted out after performing an inverse quantum Fourier transform.

Here, we first introduce the quantum Fourier transform, which will be used later in proposed protocols. The quantum Fourier transform is a linear transformation on qubits, and is the quantum version of the standard discrete Fourier transform. For 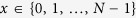, the quantum Fourier transform and the inverse quantum Fourier transform are defined as follows^17^:


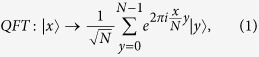



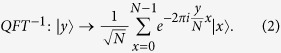


Furthermore,


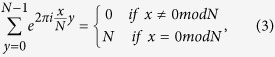


so,


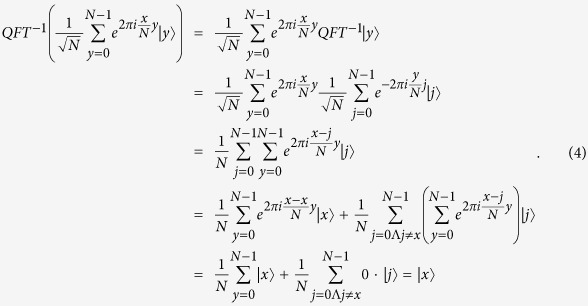


That is,


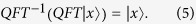


In addition, another multi-qubit quantum logic gate, which will be used later in proposed protocols, is the controlled-NOT or *CNOT* gate: 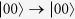, 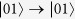, 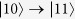 and 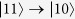, where the first qubit is the control qubit, and the second qubit is the target qubit. That is, if the control qubit is set to 0, then the target qubit is left alone. If the control qubit is set to 1, then the target qubit is flipped.

## Results

### Proposed protocols

#### Secure multiparty quantum summation

Assume that there are *n* parties: *P*_1_, *P*_2_, …, *P*_*n*_ (*n* > 2), where each party *P*_*k*_ (1 ≤ *k* ≤ *n*) has a secret integer 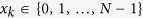 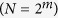, and further all *n* parties want to jointly compute the summation 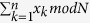 without revealing their respective secret *x*_*k*_s. In the following Protocol I, we suppose that *P*_1_ is the initiator party.

***Protocol I*** (*Secure multiparty quantum summation*)

**Step 1.** The initiator 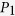 first prepares an *m*-qubit basis state 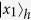, where 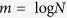 and 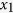 is his private secret. Then 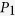 applies a quantum Fourier transform to the state 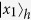 and gets the resultant state 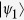. That is,


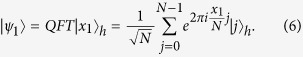


**Step 2.** 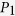 prepares another *m*-qubit ancillary state 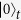 and further performs *m CNOT* gate operators on the product state 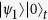, where each qubit of the first *m* qubits is the control qubit and the corresponding qubit of the second *m* qubits is the target qubit. Here we call the resultant state 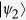, which is written as


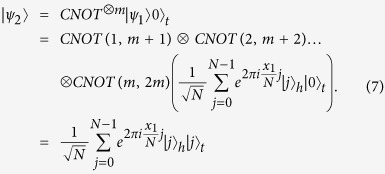


Clearly, 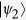 is an entangled state, where the subscript *h* or *t* denotes that the qubits will stay at home or be transmitted through the quantum channel.

**Step 3.** 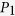 sends the second *m* qubits (i.e., the ancillary state 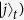 to 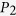 through the authenticated quantum channel.

**Step 4.** After receiving the ancillary state 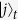, 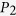 first prepares his secret state 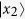. Then he applies an oracle operator 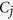 on 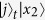, where 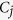 is defined by


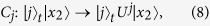


with


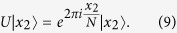


That is, 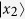 is an eigenvector of *U* with the eigenvalue 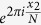. After applying the oracle operator 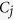, the whole composite quantum systems of 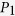 and 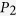 are in the following state


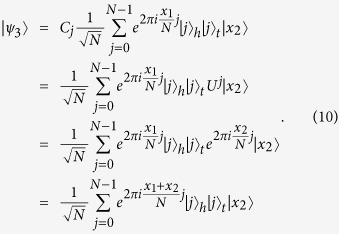


**Step 5.** Furthermore, 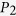 passes the ancillary state 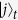 to 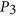 through the authenticated quantum channel and keeps 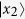 in secret. Afterward, 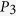 executes the similar process of 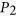, and so on. This process is repeated 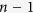 times, so that, if everyone honestly executes the protocol, the composite quantum systems of all *n* parties are in the following state


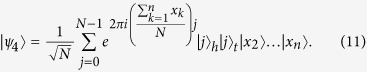


**Step 6.** Finally, 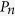 sends the ancillary state 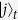 back to 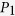. After receiving the ancillary state 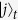, 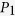 again applies 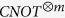 on his 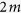 qubits, where each qubit of the first *m* qubits is the control qubit and the corresponding qubit of the second *m* qubits is the target qubit. Call the resultant state 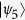. That is,


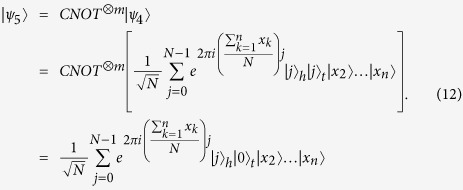


**Step 7.** Furthermore, 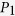 measures the second *m* qubits (i.e., 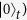 in the computational basis. If the measured result is 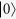, then he continues to execute the next step; otherwise he believes that there is at least one dishonest party and ends this protocol.

**Step 8.** Finally, 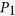 applies 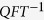 to the first *m* qubits and further measures it to obtain 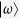, where 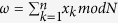.

**The correctness proof.**


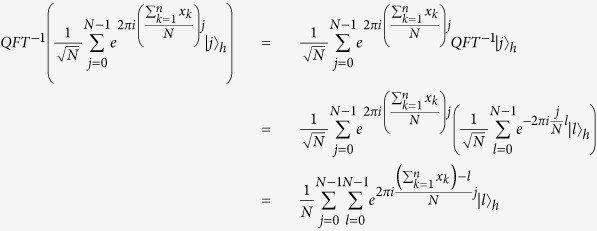



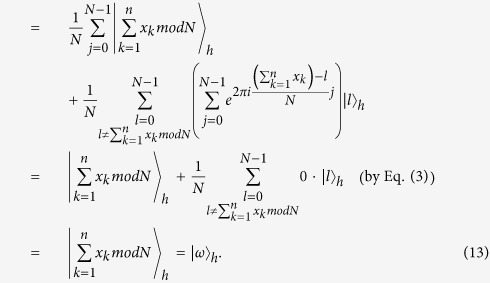


Therefore, if all parties honestly execute this protocol, 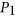 will rightly get 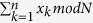.

#### Secure multiparty quantum multiplication

Assume that there are *n* parties 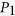, 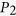, …, 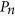 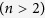, each party with a private secret 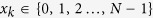 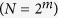, and all *n* parties want to jointly compute the multiplication of their respective private secret, i.e., 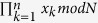. Since each secret 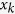 can be split and expressed as 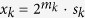, where 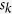 is an odd integer, then we can get


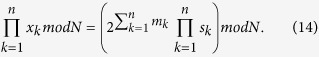


By Eq. (14), if we get the results of 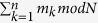 and 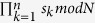, then we can easily compute 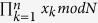. Accordingly, the computation of 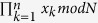 can be translated into the computations of 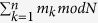 and 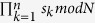, respectively. We have proposed Protocol I to compute 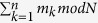. Furthermore, we present Protocol II to compute 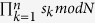, where all 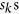 are odd integers. Similarly, in the following Protocol II, we suppose that 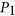 is the initiator.

***Protocol II*** (*Secure multiparty quantum multiplication*)

**Step 1.** The initiator 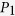 randomly chooses an odd integer 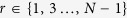 and further prepares two *m* qubits in the original state 
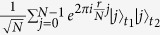
, where the preparation process is the same as that of Step 1 and 2 in Protocol I. Then 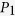 sends 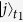 to 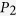 through the authenticated quantum channel and keeps 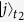 in hand.

**Step 2.** After receiving 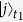, 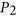 applies an oracle operator 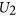 on 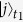 by his private secret 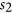, where 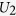 is defined by,


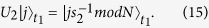


Please note that 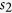 is an odd integer and 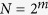, thus there exists its modulo-*N* multiplicative inverse 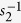, which implies that 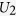 is inverse. Furthermore, 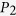 sends 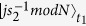 to 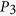 through the authenticated quantum channel. Afterward, 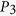 executes the similar process of 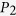 (i.e., 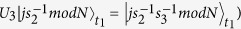, and so on. This process is repeated 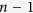 times, so that, if everyone honestly executes the protocol, the final quantum states of the qubits of the subscripts 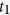 and 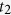 are in,


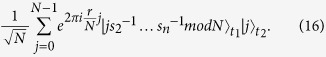


Finally, 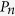 sends 
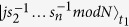
 back to 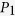.

**Step 3.** After receiving the returned state 
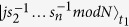
, 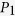 continues to send 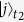 to 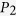 through the authenticated quantum channel.

**Step 4.** After receiving the state 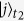, 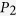 again applies the oracle operator 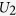 on 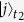 by his private input 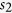, i.e., 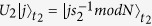. Furthermore he sends it to 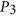 through the authenticated quantum channel, and so on. This process is repeated 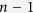 times, so that, if everyone honestly executes the protocol, the final quantum states of the 2 *m* qubits are in,


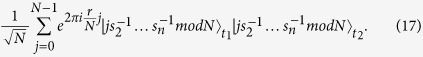


Finally, 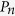 again sends 
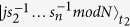
 back to 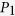.

**Step 5.** After receiving the state 
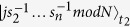
, 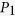 performs *m CNOT* gate operators on the two returned states, such that the quantum systems of the subscripts 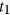 and 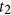 will be disentangled. That is,


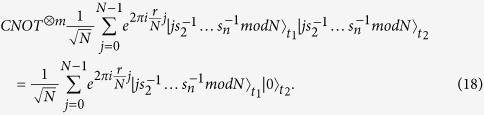


Furthermore, 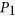 measures the qubits of the subscript 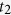 in the computation basis. If the measured result is 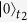, then he continues to execute the next step. Otherwise, he believes that there is at least one dishonest party and ends this protocol.

**Step 6.** Finally 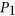 applies an inverse quantum Fourier transform 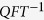 on the remaining qubits and further measures it to obtain 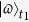 in the computation basis, where 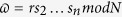. Then 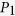 outputs 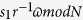. That is, 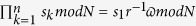.

**The correctness proof.**


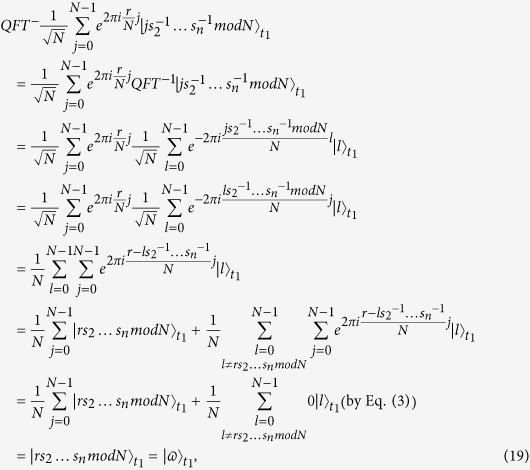


since


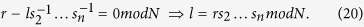


Obviously, 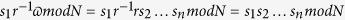, where *r* is an odd integer. Therefore, Protocol II can rightly output 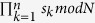. Furthermore, in order to perfectly compute 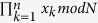, the initiator first calls Protocol I to securely compute 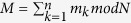 and then calls Protocol II to securely compute 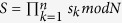. Finally, the initiator computes 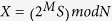. Obviously, 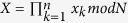.

### Security Analysis

We have analyzed the correctness of Protocol I and II, and further analyze their securities. In order to save space, please note that we mainly analyze the security of Protocol I, because the security of Protocol II is the same as that of Protocol I.

We first analyze that 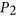 does not get any secret information about the initiator 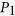
’s input 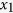. In Protocol I, 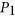 only sends the ancillary state 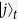 to 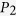 without any classical information. So, for a dishonest 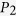, if he wants to eavesdrop 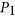
’s secret, all possible attacks he can perform with the present technology are as follows:

(1) 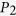 directly measures the ancillary state 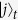 in the computational basis. Obviously, he will get 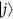 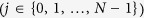 with the equal probability of 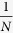, but the measured result *j* is independent of 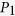
’s secret 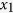. That is, this attack is infeasible.

(2) After applying a unitary operator on the ancillary state 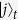, 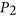 again measures it. Especially, 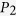 has a knowledge that 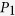
’s secret state 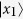 has evolved into the same state (i.e., 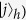 as the ancillary state 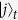 based on the quantum Fourier transform, so he may perform an inverse quantum Fourier transform 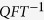 on the ancillary state 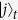 to expect to extract out 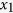. That is, this attack can be described as follows:


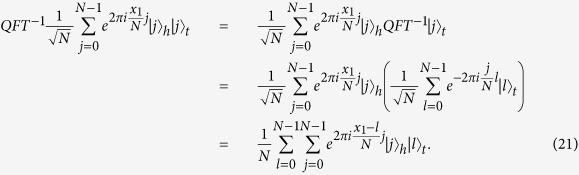


By the above equation, if 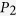 measures the ancillary state, he will get 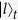 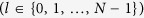 with the equal probability of 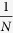. It implies that 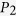 cannot get any secret information about 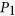
’s private input, because he cannot extract out the global phase information from the partial qubits of the entangled quantum systems with the subscripts *h* and *t*. In fact, any local unitary operator on the partial qubits cannot fully disentangle the entanglement of the composite system unless directly measured. Therefore, even if 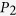 performs this attack, he still cannot get any private information about 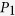
’s secret 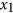.

(3) 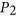 performs a more complicated entangle-measure attack that he is able to prepare another ancillary system 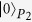 and entangle the two ancillary systems by his local unitary operations, where one is transmitted from 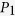, and afterward he can measure the ancillary system prepared by himself to get the partial information about 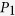
’s private inputs. 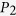
’s dishonest action when he receives 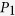
’s ancillary 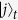 can be described by a unitary operator 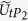, which acts on 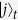 and 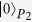. We can describe it as follows:


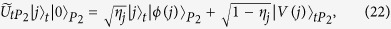


where 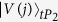 is a vector orthogonal to 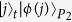, i.e.,


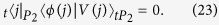


In order to completely pass the honest test (see Step 7), it can easily deduce that 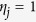. That is, the whole quantum systems of 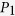 and 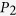 should be in the following state after performing 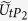:


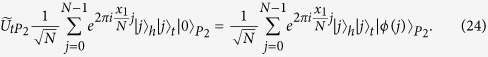


Then 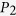 sends 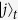 back to 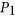. After 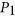 performing 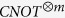 and further measuring the ancillary system *t*, the state of the remaining quantum system becomes


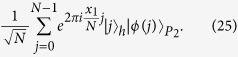


Now if 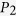 measures his ancillary state 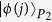, as the above analysis in the case of (2), he still cannot get any secret information about 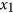 because of the entanglement of 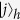 and 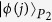. If 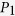 further applies 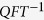 to the first *m* qubits, the state of the remaining quantum system will be updated into


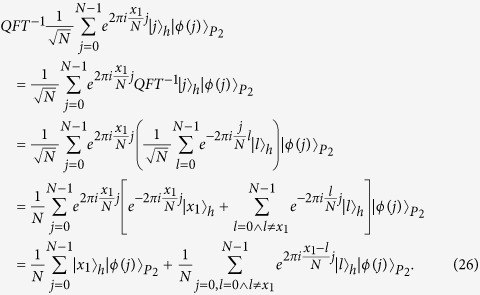


This equation shows that if 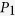 measures his remaining *m* qubits, he will get 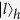 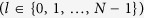 with the equal probability of 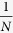, which implies that the probability of getting 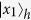 is also 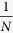, unless 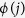 is independent of *j*. Similarly, 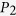 cannot get the secret 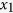 with the probability of more than 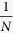 due to their entanglement yet. It implies that 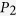 cannot get any secret information about 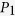
’s private input 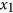. Therefore, the entangle-measure attack is infeasible.

From what we have analyzed above, we can see clearly that 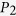 cannot get any secret information about 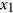. Furthermore, we can easily and naturally generalize that any party 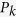 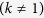 cannot obtain any secret information about 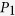
’s private input. Therefore, the initiator’s private input is unconditionally secure against other dishonest parties. In turn, if all party honesty execute this protocol, 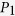 only gets the final summation 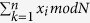 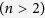, instead of single party’s private secret 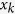. However, if the parties 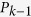 and 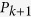 are dishonest, they can collude to get 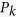
’s private input 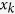. In order to overcome this weakness, we can use the communication model in a random order instead of the fixed order, that is, how to choose the next party is randomly determined by the party himself, not pre-determined by a designated party.

In addition, in order to full resist the collusion attack of any less 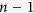 parties, we can design the following Protocol III, in which all parties are full parity.

***Protocol III*** (to compute 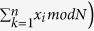


**Round 1**

**Step 1**. Each party 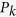 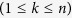 randomly generates 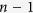 integers 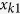, 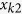, …, 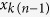 in 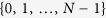, and then computes 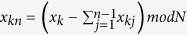. That is,


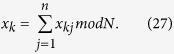


**Step 2**. Each party 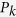 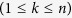 as the initiator calls Protocol I to compute


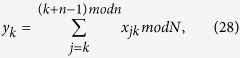


where 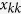 is 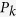
’s the initial input.

**Round 2**

Finally, all parties designate an agent who could be one of them to again call Protocol I to compute and announce


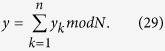


Obviously,


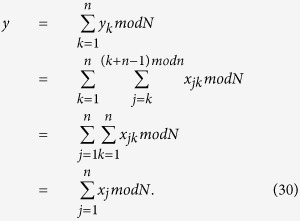


Because Protocol I can ensure the unconditional security of the private input of the initiator, every sub-secret 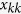 of 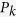 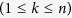 in Round 1 of Protocol III is unconditionally secure against any less 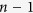 parties. Therefore, Protocol III is unconditional secure against any collusion attack, unless there are 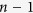 cheating parties.

As for Protocol II, obviously 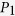
’s secret 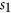 is unconditionally secure because the transmitted quantum messages don’t include any private information about 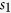. Conversely, if all parties honestly execute Protocol II, 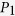 only gets the final multiplication 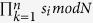 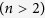, instead of certain party’s secret 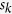. In addition, the *n*-th party 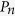 can easily perform an intercept-resend attack. That is, he intercepts all qubits passing through his hands, and then sends fake qubits back to 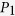. Accordingly, 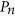 may finally obtain 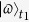 after applying *m CNOT* gate operators and an inverse quantum Fourier transform 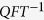 to his intercepted qubits, where 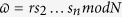. However, 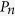 does not know *r*, so he still cannot get any secret information about other parties’ private inputs. Therefore, this attack is infeasible. Furthermore, in order to resist the collusion attack, we can also use the communication model in a random order instead of the fixed order. Similarly, we can also design the unconditionally secure quantum protocol for Secure Multiparty Multiplication.

***Protocol IV*** (to compute 
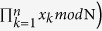


**Round 1**

**Step 1**. Each party 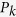 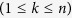 splits his secret 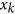 into *n* random integers 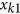, 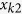, …, 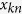 in 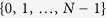, such that


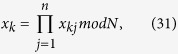


where 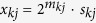. That is, 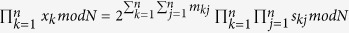.

**Step 2**. Each party 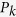 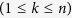 as the initiator calls Protocol III to compute


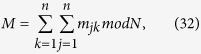


where 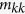 is 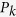
’s the initial input.

**Step 3**. At the same time, each party 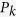 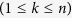 as the initiator calls Protocol II to compute


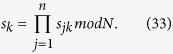


where 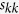 is 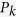
’s the initial input.

**Round 2**

Finally, all parties designate an agent who could be one of them to again call Protocol II to compute 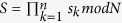 and to further announce


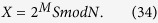


As for the security of the quantum channel, we can use the decoy technology to check eavesdropping in all proposed protocols. That is, the initiator randomly inserts enough decoy particles into the qubit sequence to be transmitted, where every decoy particle is prepared randomly with either Z-basis (i.e. 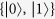 or X-basis (i.e. 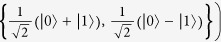. After confirming that the receiver has received the transmitted sequence, the initiator announces the positions of partial decoy particles and the corresponding measurement basis. The receiver measures these decoy particles according to the initiator’s announcements and tells the initiator his measurement results. Then the initiator compares the measurement results of the receiver with the initial states of these corresponding decoy particles in the transmitted sequence and analyzes the security of the transmissions. If the error rate is higher than the threshold determined by the channel noise, they cancel this protocol and restarts; or else they continue to the next step.

In addition, the authenticated quantum channel can further ensure the security of quantum communications. Like most existing secure multiparty quantum computations, our protocols need there is an authenticated quantum channel. This is the only assumption we need to have for proposed protocols to work. In principle, we may use a quantum authentication scheme (QAS)^18^ based on Clifford operators introduced in^19^ to implement it. We may also use quantum encryptions combined with classical authenticated keys^20^,^21^. In addition, we may still ensure the authentication by sharing the entangled quantum resources in advance^22^ or using the detecting (or decoy) particle technologies^23^.

## Discussion

In this paper, we presented a novel and efficient quantum approach to systematically compute secure multiparty summation and multiplication. In our approach, there is an initiator who prepares an entangled state and further transmits the partial qubits of the entangled state to every party in turn through the quantum channel. According to the different computations, there are two specific processing ways: the receiver in computing the summation adds his secret into the global phase of the entangled state by an oracle operator, while the receiver in computing the multiplication embeds his secret into the received basis state by another oracle operator. Finally, the initiator takes the transmitted qubits back and subtly extracts out the corresponding summation and multiplication from the phase information by an inverse quantum Fourier transform. More specifically, we proposed several quantum protocols for secure multiparty summation and multiplication, where Protocol I and II have higher efficiency due to the linear communication complexity, and Protocol III and IV provide the unconditional security and the perfect privacy protection with 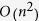 communication complexity.

In conclusion, our approach securely implements the fundamental arithmetic operations (i.e., summation and multiplication) in secret-by-secret way instead of bit-by-bit way, which may give some good references for solving other SMC problems. In theory, it can be generalized to compute lots of secure multiparty numerical computations.

## Additional Information

**How to cite this article**: Shi, R.- *et al.* Secure Multiparty Quantum Computation for Summation and Multiplication. *Sci. Rep.* **6**, 19655; doi: 10.1038/srep19655 (2016).
